# A Novel Molecular Strategy for Surveillance of Multidrug Resistant Tuberculosis in High Burden Settings

**DOI:** 10.1371/journal.pone.0146106

**Published:** 2016-01-11

**Authors:** Halima M. Said, Nicole Kushner, Shaheed V. Omar, Andries W. Dreyer, Hendrik Koornhof, Linda Erasmus, Yasmin Gardee, Ivy Rukasha, Elena Shashkina, Natalie Beylis, Gilla Kaplan, Dorothy Fallows, Nazir A. Ismail

**Affiliations:** 1 Centre for Tuberculosis, National Institute of Communicable Diseases, Sandringham, South Africa; 2 Department of Medical Microbiology, Faculty of Health Science, University of Pretoria, Pretoria, South Africa; 3 Public Health Research Institute, Rutgers University, Newark, New Jersey, United States of America; 4 National Health Laboratory Service, Groote Schuur Hospital, Cape Town, South Africa; 5 The Bill & Melinda Gates Foundation, Seattle, Washington, United States of America; University of Minnesota, UNITED STATES

## Abstract

**Background:**

In South Africa and other high prevalence countries, transmission is a significant contributor to rising rates of multidrug resistant tuberculosis (MDR-TB). Thus, there is a need to develop an early detection system for transmission clusters suitable for high burden settings. We have evaluated the discriminatory power and clustering concordance of a novel and simple genotyping approach, combining spoligotyping with *pnc*A sequencing (SpoNC), against two well-established methods: IS*6110*-RFLP and 24-loci MIRU-VNTR.

**Methods:**

A total of 216 MDR-TB isolates collected from January to June 2010 from the NHLS Central TB referral laboratory in Braamfontein, Johannesburg, representing a diversity of strains from South Africa, were included. The isolates were submitted for genotyping, *pnc*A sequencing and analysis to the Centre for Tuberculosis in South Africa and the Public Health Research Institute Tuberculosis Center at Rutgers University in the United States. Clustering rates, Hunter-Gaston Discriminatory Indexes (HGI) and Wallace coefficients were compared between the methods.

**Results:**

Overall clustering rates were high by both IS*6110*-RFLP (52.8%) and MIRU-VNTR (45.8%), indicative of on-going transmission. Both 24-loci MIRU-VNTR and IS*6110*-RFLP had similar HGI (0.972 and 0.973, respectively), with close numbers of unique profiles (87 vs. 70), clustered isolates (129 vs. 146), and cluster sizes (2 to 26 vs. 2 to 25 isolates). Spoligotyping alone was the least discriminatory (80.1% clustering, HGI 0.903), with 28 unique types. However, the discriminatory power of spoligotyping was improved when combined with *pnc*A sequencing using the SpoNC approach (61.8% clustering, HGI 0.958). A high proportion of MDR-TB isolates had mutations in *pnc*A (68%, n = 145), and *pnc*A mutations were significantly associated with clustering (p = 0.007 and p = 0.0013 by 24-loci MIRU-VNTR and IS*6110*-RFLP, respectively), suggesting high rates of resistance to pyrazinamide among all MDR-TB cases and particularly among clustered cases.

**Conclusion:**

We conclude that SpoNC provides good discrimination for MDR-TB surveillance and early identification of outbreaks in South Africa, with 24-loci MIRU-VNTR applied for *pnc*A wild-type strains as needed.

## Background

South Africa has the world’s third highest tuberculosis (TB) burden, after India and China and is second highest in terms of absolute numbers of multidrug-resistant (MDR) TB [1]. Moreover, since the 2006 Tugela Ferry outbreak of extensively drug-resistant (XDR) TB in KwaZulu-Natal, XDR-TB has been reported in all provinces in South Africa [2, 3]. Both MDR- and XDR-TB are associated with elevated mortality and high treatment costs, thus contributing a disproportionate burden on health systems [4]. The emergence of MDR-TB has been associated with inappropriate drug regimens, treatment default and HIV co-infection [5, 6]. In addition, evidence from population studies carried out in South Africa and other high prevalence settings suggest that transmission is a significant contributor to globally rising rates of MDR-TB [7–9].

The World Health Organization has set ambitious targets for TB control for the next 20 years [10]. However for these to be achieved, catalysts of transmission will need to be identified for development of targeted interventions, as discussed by Dowdy et al [11]. This would require establishment of an early warning surveillance system to detect genotypic clusters among clinical isolates of *Mycobacterium tuberculosis* (*M*. *tuberculosis*) as an indication of transmission in the population. Although routine genotyping forms an important component of TB control programs in many low prevalence settings, wide-scale genotyping in countries with high TB burden has been hampered by constrained resources and by the prevalence of endemic strains, which complicate the molecular discrimination of epidemiologically linked clusters [12].

The choice of an appropriate genotyping method depends on the extent of diversity of the biomarker among *M*. *tuberculosis* isolates in the population. The selected biomarker must be sufficiently polymorphic to distinguish unrelated strains and also stable enough to detect epidemiologically-linked strain clusters [13]. In addition, for optimal application in surveillance, the method should be rapid, reproducible, and easy to interpret in order to facilitate high-throughput analysis to limit cost and ensure timely results for public health action [14].

The use of IS*6110*-restriction fragment length polymorphism (RFLP) for TB genotyping is internationally accepted as the gold standard and provides excellent discrimination [15]. However, the method requires time for subculture to isolate high quality DNA, has limited discrimination for strains with low IS*6110* copy numbers and the results are not readily digitized, making inter-laboratory comparisons difficult [16, 17]. Polymerase chain reaction (PCR)-based mycobacterial interspersed repetitive units-variable number tandem repeats (MIRU-VNTR) typing, using 15- or 24-loci, has high discriminatory power and the results are readily compared as standardized digital codes, but this method is relatively expensive and has shown lower discrimination in settings where Beijing strains are dominant [18, 19]. Compared to IS*6110*-RFLP and MIRU-VNTR, spacer oligonucleotide typing (spoligotyping) is the simplest method to perform, yielding rapid results in the form of an internationally standardized binary pattern, making it ideal for high-throughput analysis [20]. However, spoligotyping has relatively low discriminatory power and may distort estimates of recent MDR-TB transmission by clustering endemic strains that are not necessarily epidemiologically linked [21].

A number of drug resistance mutations have been used for fine resolution of genotyping clusters [13]. The *M*. *tuberculosis pnc*A gene encodes an amidase that is required for modifying the anti-TB drug pyrazinamide (PZA) to an active state. Mutations in *pnc*A account for the majority of resistance to PZA in both clinical and laboratory isolates and show high correlation with in vitro assays of phenotypic resistance to PZA [22]. Due to the technical challenges of growth-based PZA susceptibility testing, resistance testing is not routinely performed in many high burden countries, including South Africa [23]. However, two surveys conducted in Gauteng and Western Cape between 2000 and 2002 showed that clinical isolates from over 50% of MDR-TB patients harbored mutations in the *pnc*A locus [24, 25]. This finding is evidence of a large burden of undiagnosed PZA resistance, particularly among MDR-TB cases. Importantly for its application in molecular typing, mutations in the *pnc*A locus are highly diverse, spanning a region of ~600 nt covering the entire gene and putative promoter region [26]. A recently published meta-analysis of global PZA resistance identified 608 unique polymorphisms in 397 positions throughout the *pncA* locus, with a pooled estimated prevalence of 16.2% among all TB cases and 60.5% among MDR cases [23]. Thus, the use of *pnc*A sequencing as a secondary genetic marker to improve the resolution of spoligotyping clusters offers a novel strategy to provide a high volume and faster approach for genotyping in endemic settings, with the added benefit of providing resistance information for programmatic purposes.

The present study was designed to evaluate *pnc*A sequencing as a secondary marker and to examine the association between *pnc*A mutations and clustering in an MDR-TB population in South Africa. In addition, we sought to determine the discriminatory ability and cluster concordance of spoligotyping combined with *pnc*A sequence profiling (SpoNC) compared with IS*6110*-RFLP and 24-loci MIRU genotyping methods.

## Methods

The wording of the manuscript is suitable for publication.

### Ethics

This study was approved by the Human Research Ethics Committee of the University of the Witwatersrand and the Institutional Review Board of Rutgers University. The institutional review board committee waived the need for consent.

### Clinical Samples

A total of 216 clinical isolates of *M*. *tuberculosis* from culture positive confirmed MDR-TB cases was used in this study. These isolates were collected over the first 6 months of 2010 from the NHLS Central TB referral laboratory in Braamfontein, Johannesburg. The NHLS laboratory is a high-throughput routine diagnostic laboratory that receives specimens for culture and drug susceptibility testing (DST) from over 100 health facilities in Johannesburg and surrounding areas. All isolates were submitted for genotyping and analysis to the Centre for Tuberculosis (CTB) at the National Institute for Communicable Diseases in South Africa and the Public Health Research Institute (PHRI) Tuberculosis Center at Rutgers University in the United States.

### Genotyping

Spoligotyping was done using a commercial kit (Ocimum Biosolutions, India) according to the manufacturer’s instructions [20]; spoligotypes were uploaded as binary codes in the SpolDB4 database for SIT (Spoligotype International Type) assignment. The MIRU-VNTR typing was performed using an automated 24-loci MIRU-VNTR method, as described [27], and validated using the MIRU-VNTR Calibration Kit (GenoScreen, France). The PCR products were subjected to electrophoresis using the ABI 3130XL genetic analyser (Applied Biosystems, CA, USA) with the LizMarker (Bioventures, TN, USA) as a size standard. Sizing of fragments and MIRU-VNTR allele assignation were performed using GeneMapper software 4.0 (Applied Biosystems). IS*6110*-RFLP typing was performed using the standardized methods, as described previously [15].

### Amplification and sequencing of the *pnc*A locus

Genomic DNA was prepared from all isolates and subjected to PCR amplification (primers: 5’-TGGCCGCCGCTCAGCTGGTCATG-3’; 5’-CCACCGCCGCCAACAGTTCAT-3’) followed by Sanger sequencing of *pnc*A promoter and coding DNA sequence (CDS). Mutations were identified by alignment of nucleotide sequences to MTB H37Rv reference strain (NCBI Accession number AL123456) [28] using ClustalW2 [29].

### Cluster definition

A cluster was defined for each individual method as two or more *M*. *tuberculosis* isolates with an identical genotyping pattern from different patients, while SpoNC clusters were defined as two or more isolates with an identical spoligotyping pattern and *pnc*A mutation. The clustering rate was defined as (*n*_*c*_*-c*)/*n*, where *n* is the total number of cases in the sample, *c* is the number of genotypes represented by at least two cases and *n*_*c*_ is the total number of cases in clusters of two or more patients [30].

### Statistical analysis

A chi-square test was used to determine associations between the presence of *pnc*A mutations and clustering defined by IS*6110*-RFLP or 24-loci MIRU-VNTR. The Hunter-Gaston index (HGI) was calculated as described previously [31] and used to evaluate the level of discriminatory power of the genotyping methods, alone and in combination. The HGI varies between 0.00 (where all strains in the sample are indistinguishable) and 1.00 (all strains are differentiated). Wallace coefficients are determined by calculating the ability to predict clustering generated by one typing method, given the results analysed by a second typing method and were used to measure agreement between methods, as described previously [32]. Potential values range from 0 (no ability to predict) to 100% (perfect predictive ability).

## Results

The number of IS*6110-*RFLP bands ranged from 1 to 23 per strain. Of the 216 *M*. *tuberculosis* isolates, a total of 32 clusters (146 isolates, 68%) were identified by IS*6110*-RFLP, the largest containing 25 isolates, with 70 (32%) unique isolates, giving a strain-clustering rate of 52.8% (Table 1). Of these 32 clusters, four (35 isolates, 16%) had less than six IS*6110* elements. The 24-loci MIRU-VNTR typing identified 30 clusters (129 isolates, 60%), of which the largest included 26 isolates, and 87 unique isolates (40%), giving a strain-clustering rate of 45.8%.

**Table 1:** Discriminatory power of different genotyping methods for MDR-TB isolates.

Method	N	No. of types	No. of unique isolates	No. of clustered isolates	No of clusters	Range of cluster size	HGI	Clustering rate
IS*6110*-RFLP	216	102	70	146	32	2 to 25	0.973	52.8%
24-loci MIRU-VNTR	216	117	87	129	30	2 to 26	0.972	45.8%
Spoligotyping	216	43	28	188	15	2 to 48	0.903	80.1%
Spoligotyping + *pncA* sequence*	212	81	53	159	24	2 to 29	0.958	63.7%
*excluding 4 isolates without *pnc*A sequence data								
***pncA* mutants (N = 145)**								
IS*6110*-RFLP	145	61	43	102	18	2 to 25	0.979	57.9%
24-loci MIRU-VNTR	145	73	55	90	18	2 to 24	0.984	49.7%
Spoligotyping + *pncA* mutation	145	54	37	108	17	2 to 24	0.976	62.8%

Spoligotyping resulted in 43 distinct patterns, including 28 (13%) unique and 188 (87%) clustered isolates, yielding a clustering rate of 80.1%. None of the isolates were identified as *M*. *bovis*. Among the clustered isolates, 14 strain families were identified by spoligotyping, with Beijing (48/188, 25.5%) constituting the largest, followed by LAM4 (31, 16%), H3 (26, 14%), S (18, 9.6%), EAI1_SOM, X2 and X3 (12, 6.4%), T1 (ST 53) (8, 4.3%), T1 (ST 926) (7, 3.7%), T3 (4, 2.1%), T1 (ST719), LAM3 (ST 33) and LAM3 (ST 130) (3, 1.6%), and LAM9 (2, 1.1%).

Based on HGI, the discriminatory power of 24-loci MIRU-VNTR and IS*6110*-RFLP typing were similar, while spoligotyping was the least discriminatory of the three methods (Table 1). Among the 48 Beijing strains, IS*6110*-RFLP and 24-loci MIRU-VNTR typing split the group into 8 and 9 clusters with 17 and 20 unique isolates (HGI 0.997 and 0.998), respectively. The level of concordance between typing methods was evaluated by calculation of Wallace coefficients (Table 2). Considering IS*6110*-RFLP as the reference standard, the Wallace coefficient was 78% for MIRU-VNTR and 83% for spoligotyping. When 24-loci MIRU-VNTR was used as the reference, the Wallace coefficients were somewhat higher: 88% and 87% for IS*6110*-RFLP and spoligotyping, respectively. Sixteen of the 32 clusters identified by IS*6110*-RFLP (115 isolates, 53.2%) shared fully identical genotypes by 24-loci MIRU-VNTR typing. Three IS6110-RFLP clusters were split partially by 24-loci MIRU-VNTR typing, while the remaining 7 clusters were fully differentiated. Conversely, 9 clusters defined by 24-loci MIRU-VNTR typing were partially split by IS*6110*-RFLP typing and 5 clusters were fully differentiated. Taken together, the similarities in discriminatory power and clustering rates and the high concordance between the two typing methods support the validity of 24-loci MIRU-VNTR genotyping, despite the high proportion of Beijing strains in the population.

**Table 2:** Concordance between the typing methods in total population (N = 216).

Reference typing method	Complementary method	Wallace coefficient
IS*6110*-RFLP	24-loci MIRU-VNTR	78.2
IS*6110*-RFLP	Spoligotyping	82.8
24-loci MIRU-VNTR	IS*6110*-RFLP	87.8
24-loci MIRU-VNTR	Spoligotyping	87.4
Spoligotyping	IS*6110*-RFLP	70.6
Spoligotyping	24-loci MIRU-VNTR	60.6

### Use of *pnc*A sequence as a secondary marker and association with clustering

In our study population, 145 isolates (68%) had mutations in the *pnc*A locus, while 67 isolates had wild type *pnc*A sequence. For four isolates, *pnc*A sequencing results were not available. Diversity at the *pnc*A locus was high, with 47 unique mutations identified, including 5 insertions, 6 deletions, 35 non-synonymous single nucleotide polymorphisms (SNPs) and one synonymous SNP. The frequency of *pnc*A mutations was higher among isolates clustered by both IS*6110*-RFLP (102/133, 77%) and MIRU-VNTR (98/126, 78%) versus non-clustered isolates (43/79, 54% and 47/86, 54.7%; respectively), demonstrating a significant association between *pnc*A mutations and clustering (*p* = 0.0013, *p* = 0.0007; respectively).

The ability of *pnc*A sequencing to further resolve clusters defined by each of the primary typing methods was examined among the 145 isolates with *pnc*A mutations. Nine IS*6110*-RFLP-defined clusters (51 isolates) and 12 MIRU-VNTR-defined clusters (54 isolates) harbored identical mutations in the *pnc*A locus unique to each cluster. Fifteen IS*6110*-RFLP and 11 MIRU-VNTR clusters were subdivided by *pnc*A sequence, increasing the HGI of IS*6110*-RFLP and MIRU-VNTR to 0.979 and 0.984, respectively (Table 1).

### Performance of the SpoNC strategy

Spoligotyping in combination with *pnc*A sequence profiles defined 24 clusters, ranging in size from 2 to 29 members, with 53 unique isolates. With the exception of one cluster, *pnc*A sequence patterns subdivided all spoligotyping clusters, reducing the clustering rate from 80.1% to 63.7% and increasing the discriminatory power from 0.903 to 0.958 (Table 1). The ability of *pncA* sequencing to improve the discriminatory power of spoligotyping was even more striking when the analysis was limited to those isolates with *pncA* mutations, resulting in an HGI increased to 0.976 and a clustering rate of 62.8% (Table 1). Furthermore, the concordance was similar when assessing the new strategy with either IS*6110*-RFLP or 24-loci MIRU-VNTR as the reference standard and was consistently within the same range when the reference standards were compared against each other (Tables 2 and 3). Interestingly, while diverse *pnc*A mutations were able to further resolve clusters defined by each of the three primary typing methods, there were no instances where identical *pnc*A sequences were found to be present in more than one cluster defined by IS*6110*-RFLP. There was only one instance where the same *pnc*A mutation was present in two isolates with different spoligotypes (LAM3 and LAM4). Four distinct *pnc*A mutations were present in more than one 24-loci MIRU-VNTR cluster, but in all cases, the clusters differed by only a single MIRU-VNTR locus and were thus closely related. Table 4 shows the 16 clusters defined by SpoNC. Each of these clusters is defined by a unique non-synonymous SNP in the *pnc*A locus, and 11 of these specific combinations of spoligotype and *pncA* mutation have been described in previous reports from South Africa (Table 4).

**Table 3:** Concordance between the typing methods in strains with *pncA* mutation (N = 145).

Reference typing method	Complementary method	Wallace coefficient
IS*6110*-RFLP	Spoligotyping + *pncA*	88.1
Spoligotyping + *pncA*	IS*6110*-RFLP	87.2
24-loci MIRU-VNTR	Spoligotyping + *pncA*	87.3
Spoligotyping + *pncA*	24-loci MIRU-VNTR	83.8

**Table 4:** SpoNC clusters.

Spoligotype	ST	*pncA* SNP	*pncA* nucleotide position	*pncA* SNP result	Cluster size	References
		T>C	40	C14R	10	[6], [25]
BEIJING	1	ins G	518	frame shift	2	[6]
		C>G	200	S67W	2	
		ins C	456	frame shift	10	[39]
LAM4	60	del T	389	frame shift	8	
		G>C	395	G132A	3	[2]
		T>G	416	V139G	5	
H3	50	C>T	211	H71Y	24	[25], [24]
		C>T	305	A102V	3	[25]
S	34	C>A	401	A134D	3	
		T>G	100	Y34D	2	
X3	92	A>G	460	R154G	10	[25]
X2	18	G>T	289	G97C	9	[25], [43]
EAI1_SOM	48	G>A	289	G97D	7	[25]
T1	926	T>C	452	L151S	6	[25]
T3	37	T>C	175	S59P	4	[25]

## Discussion

In our study population, 68% of all MDR-TB strains were clustered by IS*6110*-RFLP typing and 60% were clustered by 24-loci MIRU-VNTR. Similarly high levels of clustering among MDR-TB cases have been described in previous studies from South Africa [33–35], consistent with transmission as a significant contributor to the growing MDR burden. These findings highlight the need for an early warning surveillance system based on genotyping of clinical strains from MDR-TB patients. The relative distribution of spoligotypes in our study population was similar to that reported in a previous study of clinical isolates collected from 434 MDR-TB cases in Gauteng from March 2004 to December 2007 [34]. However, our data suggest that there were increases in the relative proportions of Beijing and H3 (ST 50) MDR strains in the Gauteng MDR population between 2007 and 2010. Routine genotyping of MDR-TB strains will help in tracking such shifts in the population, which may reflect transmission and/or migration of MDR-TB within and between different regions in South Africa.

A large proportion of isolates (68%) in the MDR population carried mutations in the *pnc*A locus, consistent with other reports from South Africa [24, 25]. While not all *pnc*A polymorphisms confer drug resistance, a strong association between *pnc*A mutations and phenotypic PZA resistance has been found in clinical isolates from diverse settings [36, 37]. Of the 16 *pnc*A mutations found among the clustered isolates shown in Table 4, ten have been reported to confer PZA resistance [23]. The strong association between PZA resistance and *pnc*A mutations supports an additional benefit of routine *pnc*A sequencing, which can provide a rough estimate of PZA resistance in the MDR population and may be of value in designing protocols for improved clinical management.

As expected, a high degree of diversity among *pnc*A mutations was observed. Moreover, we found a significant association between mutations in *pnc*A and clustering by both IS*6110*-RFLP and 24-loci MIRU-VNTR, consistent with a recent report on PZA resistance among TB cases in New York City [38]. Although only 60% of clinical MDR isolates in our study population carried mutations in *pnc*A, the cluster analysis indicates that our SpoNC typing approach will enable us to follow the largest clusters of MDR-TB cases. Thus, while problematic from a public health perspective, the elevated frequency of *pnc*A mutations among clustered isolates may enhance our ability to identify possible chains of transmission and conduct outbreak investigations.

The use of *pnc*A sequencing as a secondary marker improved the discriminatory power of each of the three genotyping methods. In particular, *pnc*A sequence profiles reduced the clustering rate of spoligotyping from 80% to 62%, and yielded the highest Wallace coefficient when either IS*6110*-RFLP or MIRU-VNTR was used as a reference. Among the 16 clusters defined by spoligotyping and *pnc*A sequencing, eleven carried distinct non-synonymous SNPs that were identical to *pnc*A mutations previously seen in MDR-TB isolates from South Africa (Table 4). Two of these SNPs (T>C in position 40 and insertion G in position 518) were identified in association with a large cluster of Beijing strains predominantly from Eastern Cape [6]. Two other mutations (insertion C in position 456 and G>C in position 395) have been observed among a group of closely related LAM4/KZN strains that were originally associated with the 2006 Tugela Ferry outbreak and appear to be spreading in South Africa [2, 39]. The presence of identical *pnc*A sequences in clustered strains may be indicative of clonal strains of MDR *M*. *tuberculosis* circulating in the population of Gauteng, through transmission and/or migration of people into the region.

The discriminatory power of the SpoNC strategy was similar to IS*6110*-RFLP and 24-loci MIRU-VNTR typing, and was able to detect important clonal strains that are relevant to the South African context. This strategy is a simple high-throughput method and results can easily be interpreted and readily be compared between different laboratories. Although genomic DNA was amplified for *pncA* sequencing in the present study, a recent report describes a method for sequencing of *pncA* directly from clinical specimens that shows high sensitivity and specificity [40]. Thus, unlike IS*6110*-RFLP, SpoNC can be done directly from clinical samples without the need for prior culture, as well as from Ziehl-Neelsen smear slides. Importantly, the turnaround time for both SpoNC (1–10) and MIRU-VNTR typing (2–10 days) are greatly reduced compared to IS*6110*-RFLP (20–40 days). Moreover, SpoNC is less costly in terms of consumables and labor as compared to either IS*6110*-RFLP or 24-loci MIRU-VNTR typing. Taken together, the results of this study support the value of SpoNC strategy for MDR-TB surveillance in a high burden setting. Based on our findings, we propose to employ a tiered approach for MDR-TB transmission surveillance, involving SpoNC strategy as a first-line method, followed where relevant by 24-loci MIRU-VNTR typing of isolates that carry wild type *pnc*A sequence and are not uniquely differentiated by spoligotyping alone. MIRU-VNTR typing has a technical advantage over IS*6110*-RFLP and, despite the relatively high proportion of Beijing isolates in our population, we found 24-loci MIRU-VNTR as discriminatory as IS*6110*-RFLP for genotyping. These findings support the use of 24-loci MIRU-VNTR for genotyping isolates with wild type *pnc*A sequences and non-unique spoligotypes. However the follow on method would be applied on risk or need basis, since within this group that are *pnc*A wild type the cluster sizes were small between 2–4 and these small clusters may not be an early priority in high burden settings where emphasis would be on targeting the large clusters with greater risk potential. This would simplify the approach and ensure resources are directed where the need is greatest.

A limitation of the present study is the lack of epidemiological and contact tracing data to validate and interpret clusters identified by the three typing methods. However, we have used both IS*6110*-RFLP and 24-loci MIRU-VNTR as reference standards, which have been widely used in contact tracing and epidemiological investigations. In addition, the collection of isolates may not be fully representative of the MDR-TB population in South Africa, where regional diversity in *M*. *tuberculosis* strain populations has been noted [41, 42]. Further work is planned to evaluate the performance of this new strategy in other regions of South Africa to address this short coming. It should also be noted that, although the strains in this study were only from one region in South Africa, this collection identified clusters representing all the major clones present in the country

In conclusion, spoligotyping in combination with *pnc*A sequencing is an appropriate method for first-line approach for the detection of MDR-TB genotypic clusters in South Africa and similar settings, with complementary genotyping by 24-loci MIRU-VNTR, as needed. The results of this study further support the need for implementation of prospective MDR-TB transmission surveillance for early identification of outbreaks. Lastly, molecular surveillance at a national level will provide insights into MDR-TB transmission that can inform targeted interventions to control spread and reduce incidence of MDR-TB in South Africa.

S1 File. Typing methods used and gen sequenced for each patient.

“IS6110-RFLP” = Insertion sequence 6110 restriction fragment length polymorphism; “pncA” = gene sequenced for Pyrazinamide resistance; “ST” = shared type; “MIRU-VNTR” = Mycobacterial Interspersed Repetitive Unit-Variable Number tandem repeats

## Supporting Information

S1 File (XLSX)
